# Supplementary material for: Topological properties and organizing principles of semantic networks
Source: Sci Rep. 2023 Jul 20;13:11728. doi: 10.1038/s41598-023-37294-8 (PMC10359341; doi:10.1038/s41598-023-37294-8)
Supplement: Supplementary file 1 — Supplementary Information. [file 41598_2023_37294_MOESM1_ESM.pdf]

Topological properties and organizing principles of semantic networks

Gabriel Budel[1,*,+], Ying Jin[1,+], Piet Van Mieghem[1], and Maksim Kitsak[1]

[1]Faculty of Electrical Engineering, Mathematics and Computer Science, Delft University of Technology, 2628 CD, Delft, Netherlands
[*]G.J.A.Budel@tudelft.nl
[+]these authors contributed equally to this work

Supplementary Information

Data

ConceptNet[1] is a multilingual database in the form of a semantic network where nodes are words and phrases from natural language. The links indicate in total 34 different semantic relations. The 'knowledge' is collected from a variety of resources, including crowdsourced resources, expert-created resources, and games with a purpose[1]. We study the semantic networks from ConceptNet as it is one of the richest semantic network resources available.

We study the networks belonging to in total 7 link types (relations), 6 of which are directly contained within ConceptNet. They are the 'Has-A', 'Part-Of', 'Is-A', 'Related-To', 'Antonym' and 'Synonym' relations. These are the relation types in ConceptNet that we deem the most meaningful and which also have a sufficient amount of data. In addition, we define an additional link type 'Union', which is the set union of the nodes and links of four networks: 'Has-A', 'Part-Of', 'Is-A' and 'Related-To'. The purpose of adding this link type is to treat all four relations equally and to evaluate how the structure of the whole network is different from the individual ones. The definitions of the six selected relations from ConceptNet and related examples are outlined in Table S1. The links of some networks are directed, *i.e.*, of the 'Has-A', 'Part-Of', and 'Is-A' networks, but we treat all networks as undirected for simplicity of the analysis and also comparability. We remove nodes with phrases consisting of more than 5 words, as we deem these to be artifacts of the automated data extraction in ConceptNet.

Semantic relations

Relation	*Description*	*Directed*	*Example*	*Creation Method*
Has-A	B belongs to A, either as an inherent part or due to a social construct of possession. Has-A is often the reverse of Part-Of.	Yes	bird $\rightarrow$ wing	Manual + Automatic
Part-Of	A is a part of B. This is the part meronym relation in WordNet.	Yes	gearshift $\rightarrow$ car	Manual + Automatic
Is-A	A is a subtype or a specific instance of B; every A is a B. This can include specific instances; the distinction between subtypes and instances is often blurry in language. This is the hyponym relation in WordNet.	Yes	car $\rightarrow$ vehicle	Manual + Automatic
Related-To	The most general relation. There is some positive relationship between A and B, but ConceptNet can't determine what that relationship is based on the data.	No	learn $\leftrightarrow$ erudition	Manual + Automatic
Antonym	A and B are opposites in some relevant way, such as being opposite ends of a scale, or fundamentally similar things with a key difference between them. Counterintuitively, two concepts must be quite similar before people consider them antonyms. This is the antonym relation in WordNet.	No	black $\leftrightarrow$ white	Automatic
Synonym	A and B have very similar meanings. They may be translations of each other in different languages. This is the synonym relation in WordNet.	No	sunlight $\leftrightarrow$ sunshine	Automatic

Table S1. Definition of the six relations and related information from ConceptNet[2].

Overview of the English semantic networks

In Table S2, we list basic descriptive statistics of the seven semantic networks: the number of nodes N, the number of links L, the maximum degree d_{max} and the average degree $E[D]$.

Based on the number of nodes, network 'Has-A' is the smallest ($N = 7,503$) and network 'Union' is the largest ($N = 677,426$). The number of links L ranges from 5,421 to 1,819,646. Relative to the network sizes, all 7 networks have a small average degree, ranging from 1.45 to 5.43. For instance, in network 'Part-Of', on average a node only has connections to 2 (0.02%) of the in total 11,839 nodes. In other words, the number of links is of the same order as the number of nodes, which indicates that semantic networks are sparse.

Network	***Has-A***	***Is-A***	***Part-Of***	***Related-To***	***Union***	***Antonym***	***Synonym***
N	7,503	152,538	11,839	592,816	677,426	16,867	166,922
L	5,421	220,589	12,003	1,610,452	1,819,646	14,371	155,048
d_{max}	372	2913	116	4025	5263	38	103
$E[D]$	1.45	2.89	2.03	5.43	5.37	1.70	1.86

Table S2. Basic statistics of the seven English semantic networks extracted from ConceptNet.

Descriptive statistics semantic networks

Table S3 shows the overall descriptive statistics of the English semantic networks: the number of nodes N, the number of links L, the maximum degree d_{max}, the average degree $E[D]$, the average nearest neighbor degree (ANND), the graph clustering coefficient c_G and the estimated power-law exponents $\hat{\gamma}$. We rewire all semantic networks using the methods described before, after which the same statistics are calculated for the rewired networks.

For networks obtained by degree-preserving rewiring, only the ANND and the graph clustering coefficient c_G change. The average nearest neighbor degree ANND becomes smaller for all randomized semantic networks, except for the 'Synonym' network.

All networks except the 'Has-A' network have a remarkably larger graph clustering coefficient c_G (at least by an order of magnitude) than the randomized networks. Because in random networks links are randomly distributed, there are fewer triangles. On the contrary, the randomized networks of 'Has-A' exhibit a clustering coefficient more than seven times larger than their original networks.

Network	***Has-A***	***Is-A***	***Part-Of***	***Related-To***	***Union***	***Antonym***	***Synonym***
N	1,664	140,024	7,562	571,079	650,079	5,912	53,279
L	1,842	213,319	9,212	1,598,548	1,804,666	7,986	80,668
d_{max}	198	2913	116	4025	5263	38	103
$E[D]$	2.21	3.05	2.44	5.60	5.55	2.70	3.03
ANND	33.6	242	14.1	170	219	6.77	7.13
ANND rewired	23.3	142	10.8	145	173	6.25	7.51
c_G	2.17×10^{-3}	5.66×10^{-2}	4.61×10^{-2}	1.02×10^{-1}	1.04×10^{-1}	1.50×10^{-2}	1.13×10^{-1}
c_G *rewired*	1.83×10^{-2}	6.26×10^{-3}	1.95×10^{-3}	3.26×10^{-3}	3.68×10^{-3}	7.26×10^{-4}	1.48×10^{-4}
$\hat{\gamma}^{Slope}$	2.3	2.3	2.4	2.4	2.4	$\times$	$\times$
$\hat{\gamma}^{Hill}$	2.3	2.3	2.5	2.3	2.3	$\times$	$\times$
$\hat{\gamma}^{Mom}$	2.5	2.3	2.6	2.2	2.2	$\times$	$\times$
$\hat{\gamma}^{Kern}$	2.6	2.3	2.7	2.1	2.1	$\times$	$\times$

Table S3. Statistics of the LCCs of seven English semantic networks extracted from ConceptNet. A cross ($\times$) indicates the degree sequence of the corresponding network is hardly or no power-law.

In summary, we find universalities across semantic networks from different languages in the *degree distribution*, *degree assortativity*, *clustering*, *sparsity* and *connectedness*. Most semantic networks have power-law degree distributions and most of them are scale-free networks. There are two types of degree mixing patterns in semantic networks: assortative and disassortative. Most networks have higher average clustering coefficients than expected by chance, except for one network, the network 'Has-A', which shows lower clustering. All semantic networks have high sparsity. Most networks have a single connected component containing the majority of the nodes, except for the network 'Has-A', which is more fragmented.

Number of nodes in the LCCs of the English networks

Network	Size of full network	Number of nodes in LCC	Percentage
Has-A	7,503	1,664	22.18%
Has-A (rewired)		2,416 ± 35	(32.20 ± 0.47)%
Is-A	152,538	140,024	91.80%
Is-A (rewired)		127,258 ± 73	(83.43 ± 0.05)%
Part-Of	11,839	7,562	63.87%
Part-Of (rewired)		7,993 ± 53	(67.51 ± 0.45)%
Related-To	592,816	571,079	96.33%
Related-To (rewired)		570,012 ± 116	(96.15 ± 0.02)%
Union	677,426	650,079	95.96%
Union (rewired)		650,474 ± 182	(95.77 ± 0.03)%
Antonym	16,867	5,912	35.05%
Antonym (rewired)		8,845 ± 59	(52.44 ± 0.35)%
Synonym	166,922	53,279	31.92%
Synonym (rewired)		103,466 ± 142	(61.98 ± 0.09)%

Table S4. Number of nodes in the LCCs of the seven English networks in the original and rewired networks. The LCC sizes of the rewired networks are each the average over 10 rewiring realizations with standard deviation shown.

Degree-preserving network rewiring

Degree-preserving network rewiring randomly rewires the links between nodes without changing the node degrees. To preserve the degrees of all nodes, we randomly select 1 link pair (4 nodes) and swap the endpoints of these 2 links. Figure S1 illustrates the rewiring method. To make sure that all links are likely to be rewired at least once, we repeat the random selection of links for T times, where we choose $T = 4L$, four times the number of links. The pseudocode is provided in Algorithm 1.

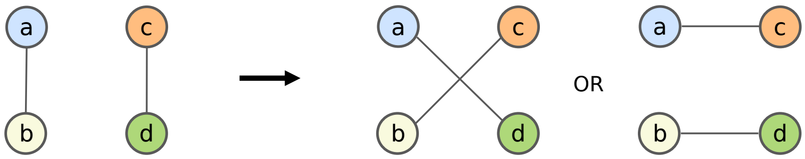

Figure S1. Illustration of degree-preserving rewiring. By randomly swapping the endpoints of two links (a,b) and (c,d), new links can be constructed without changing the node degrees.

Algorithm 1: Degree-preserving network rewiring

```
Data: a list of links
Result: a rewired network
E ← a list of links;
T ← 4L ;                                   /* all links are rewired at least once */
while T ≠ 0 do
    (a,b) and (c,d) ← randomly pick 2 links from E;
    n ← |set(a,b,c,d)| ;                   /* number of unique nodes in 2 links */
    if n < 4 then
        continue
    else
        a and c ← randomly select one node from each link ;
        (c,b) and (a,d) ← swap the two selected nodes;
        if (c,b) ∈ E or (a,d) ∈ E then
            continue
        else
            E ← update the list of links with the 2 rewired links (c,b) and (a,d);
            T ← T − 1
        end
    end
end
```

Distribution of connected components

We compute all connected components for each network and count the occurrence of the different component sizes. The results are presented in Figure S2. Overall, almost every network has a large connected component that is several orders of magnitude larger than the other connected components, except for the network ‘Has-A’, which has multiple larger connected components. Hence, network ‘Has-A’ is more fragmented, having three relatively larger connected components, where the node with the largest degree is not in the LCC but in the second largest one. We inspected each of these three connected components and find that each of the components has a distinct theme. For example, the component with the largest degree node contains all kinds of disease names. We believe that the fragmentation is caused by the partial automatic creation of the dataset.

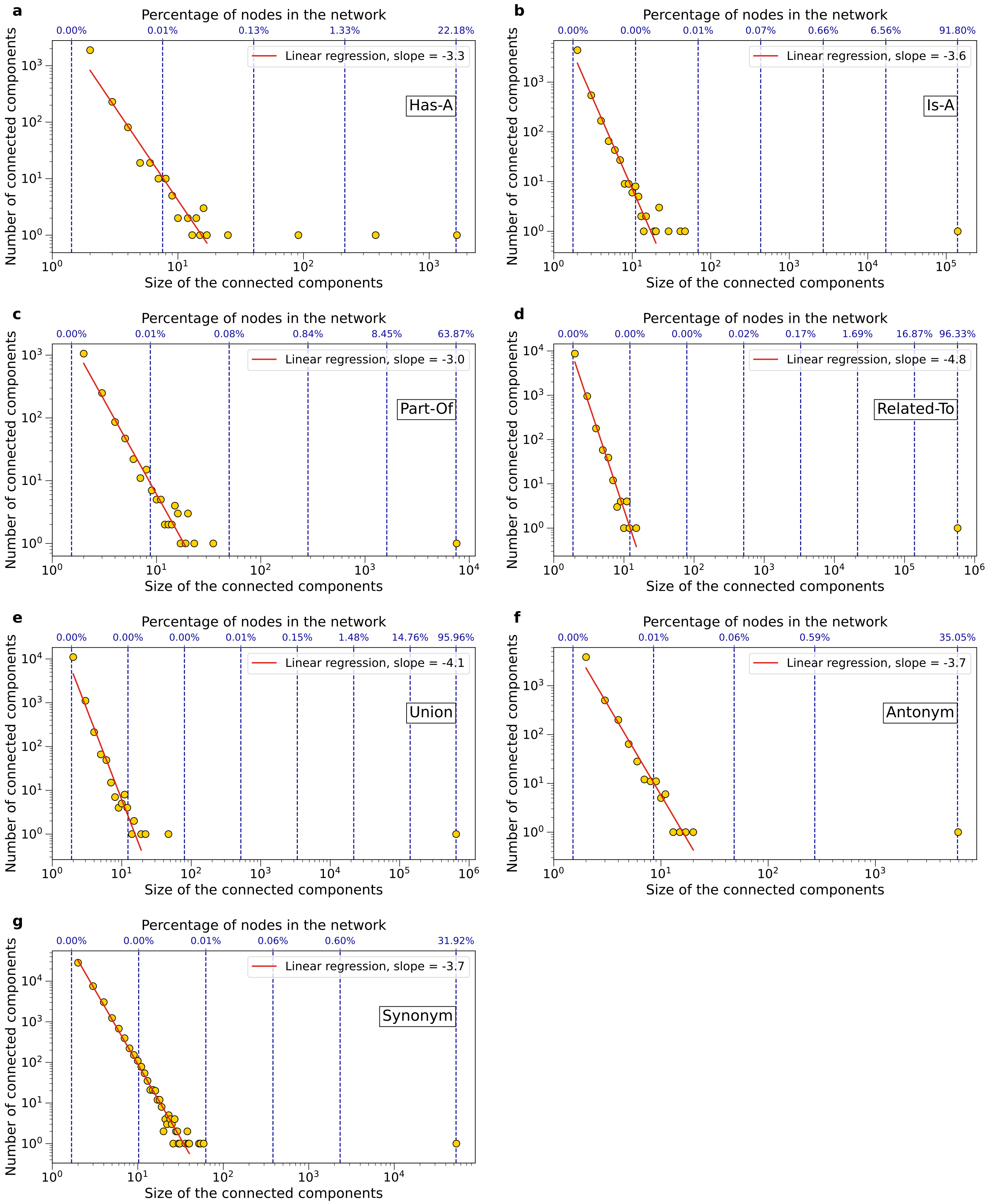

Figure S2. Size distributions of connected components of the seven English semantic networks. The dashed lines indicate the percentage of nodes in the connected components relative to the network total. The regression line is fitted on the bulk of the data points.

Power-law degree distributions

Logarithmic binning

To suppress noise at larger values of the degree k in the density of the degree distribution $\Pr[D=k]$ (the tail), we group the data in bins of equal logarithmic width. In linear binning, every bin has the same linear width $w = k_{i+1} - k_i$, while in logarithmic binning, the bins have constant logarithmic width b, where $b = \log(k_{i+1}) - \log(k_i)$[3]. Thus, the linear bin width of a logarithmic bin, $w_i = k_{i+1} - k_i = k_i(e^b - 1)$, is proportional to k_i. The sizes of the logarithmic bins grow exponentially. Therefore, the number of observations x in a bin is equal to the density of observations $f(k)$ in that bin times the width w of that bin.

Simple power-law exponent estimation

A common method for estimating the power-law exponent γ in $\Pr[D=k] \approx ck^{-\gamma}$ is to measure the slope of $\log(\Pr[D=k])$ versus $\log(k)$. Since the probability density function $f(k)$ of the degree is proportional to $k^{-\gamma}$, the number of observations $x \propto f(k) \times w \propto k^{1-\gamma}$. Regressing $\log(x)$ against $\log(k)$ yields a slope equal to $1-\gamma$. To estimate γ, normalization of the number of observations x is required. Due to the increasing bin width, a bin can contain more than one value of k. The sum of all observations within a bin is x. To preserve the probability of a node with degree k such that the total probability of degree distribution is equal to 1, the number of observations x is normalized by the linear width of the bin. This converts x to the number of observations per unit of the bin width, $(x/w) \propto k^{-\gamma}$. As a result, regressing the normalized logarithmic bin counts $\log(x/w)$ against the logarithmic degree $\log(k)$ yields a slope of $-\gamma$[3]. We base our slope estimate of γ on the linear part of the tail in the density $\Pr[D=k]$, which we determine by inspection for each network.

Consistent power-law exponent estimators

A more rigorous approach to power-law degree distributions is provided by Voitalov *et al.*[4], here we summarize the main conclusions for the convenience of the reader. They consider a degree distribution to be a power law if the probability density function is a member of the class of regularly varying functions: $\Pr[D=k] = \ell(k)k^{-\gamma}$, where $\ell(k)$ is a slowly varying function. The function $\ell(k)$ is called slowly varying if

$$\lim_{k\to\infty} \frac{\ell(ak)}{\ell(k)} = 1, \tag{1}$$

for any $a > 0$. This definition corresponds with a perfect power law in the tail of the distribution. Voitalov *et al.* propose to use three different consistent estimators of the power-law exponent: the Hill, moments and kernel estimators. We use their software package to obtain these estimates for the degree sequences of our networks[5]. These estimators do not estimate γ directly, but rather the extreme value index

$$\xi = \frac{1}{\gamma - 1}. \tag{2}$$

As a rule of thumb, Voitalov *et al.* consider a distribution to be a power law if $\hat{\xi} > 1/4$ for all three estimators[4], corresponding with $\hat{\gamma} < 5$. Here, we adopt this rule for distinguishing power-law distributions. In addition, they call a distribution *hardly power-law* if all $\hat{\xi} > 0$, but at least one $\hat{\xi} \leq 1/4$ ($\hat{\gamma} > 5$). If any $\hat{\xi} < 0$, the distribution is not a power law.

Average nearest neighbor degree as a function of the degree

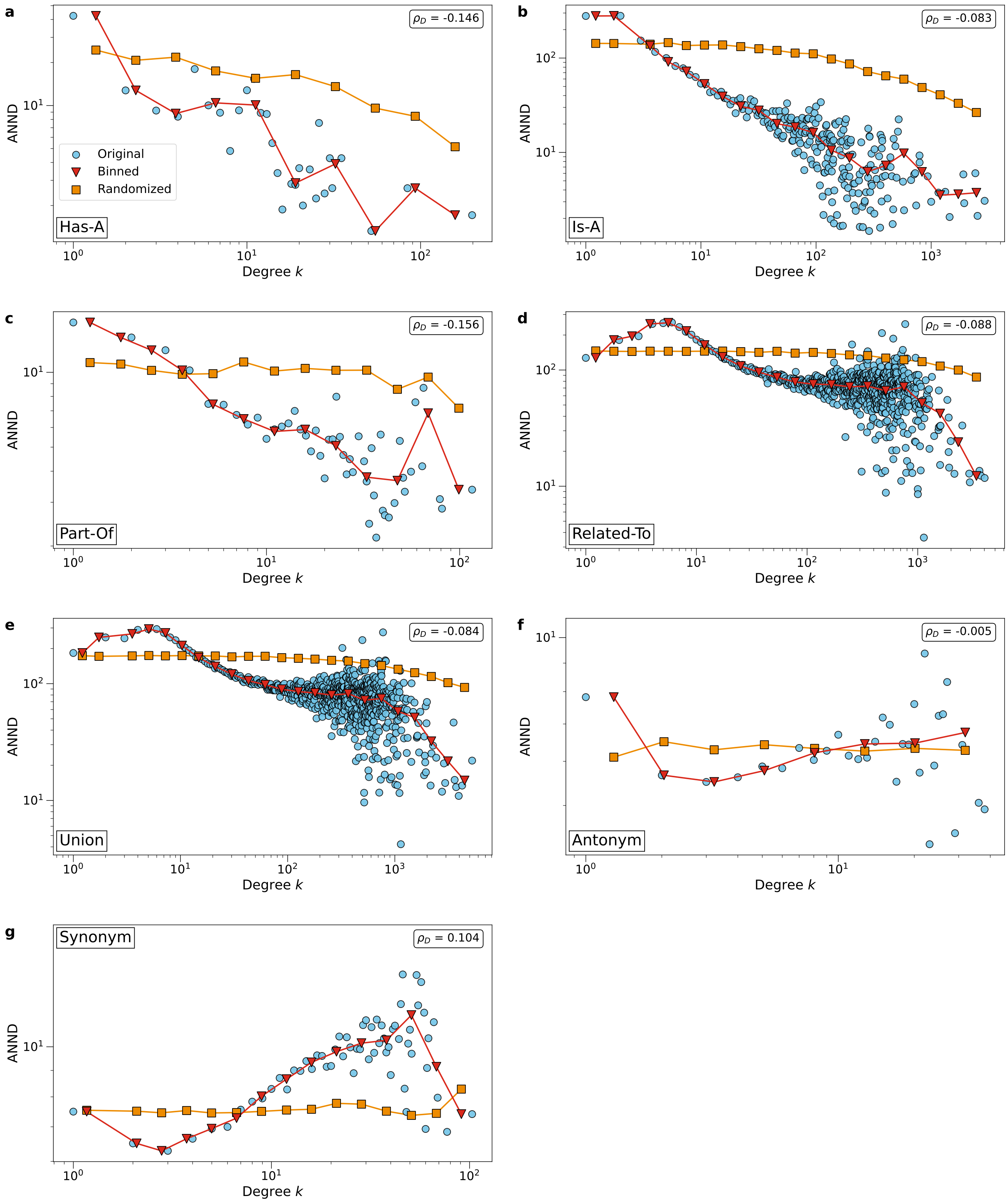

Figure S3. Average nearest neighbor degree (ANND) as a function of the degree k and degree correlation coefficient ρ_D of the seven English semantic networks. (a) Network *'Has-A'*, (b) Network *'Is-A'*, (c) Network *'Part-Of'*, (d) Network *'Related-To'*, (e) Network *'Union'*, (f) Network *'Antonym'*, (g) Network *'Synonym'*. The circle data points are the original average ANND of nodes with degree k in a network, triangles represent the data after logarithmic binning, and squares are the average ANND of nodes with degree k in the randomized network.

Clustering coefficient as a function of degree

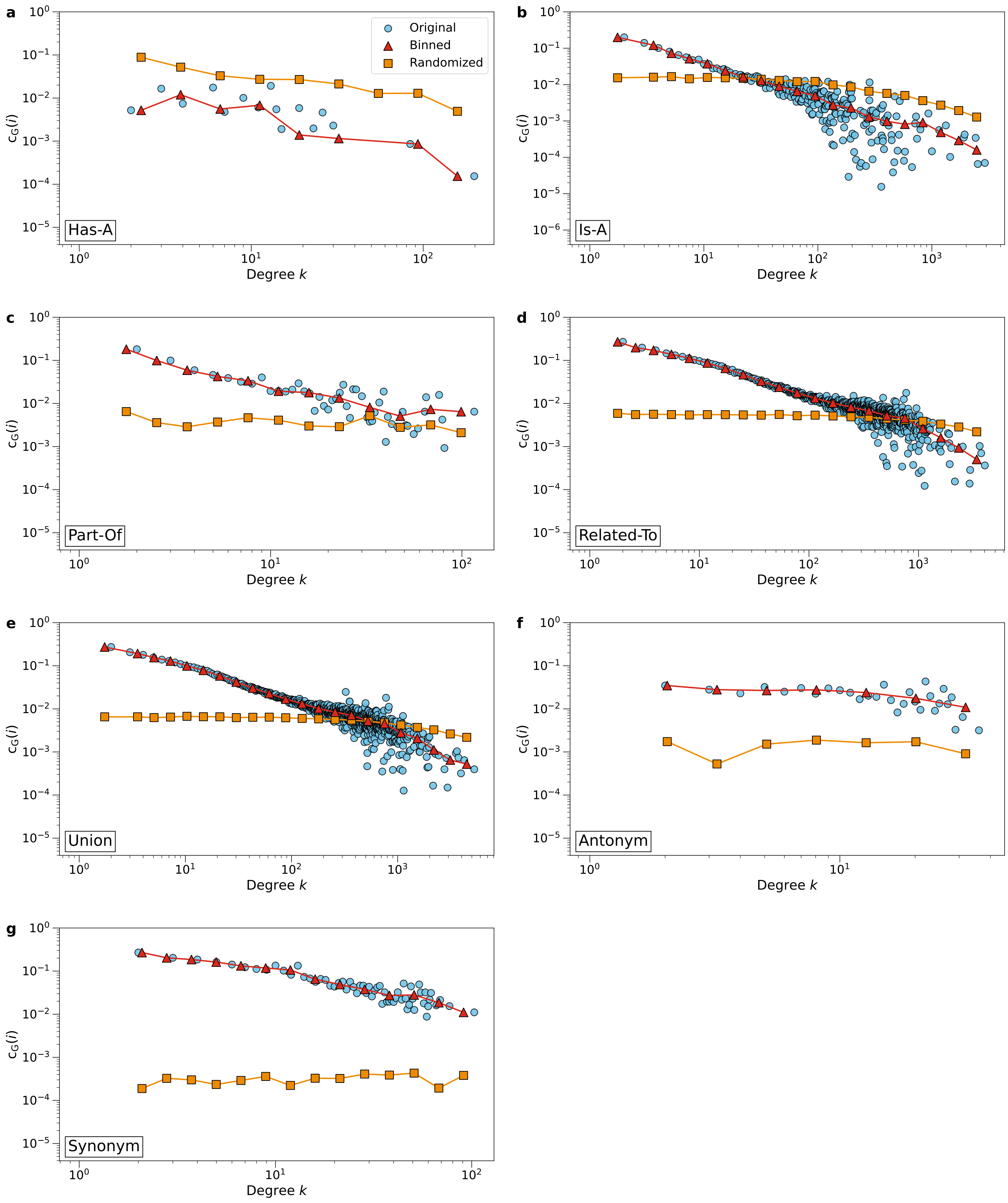

Figure S4. The average clustering coefficient $c_G(i)$ of nodes with degree $d_i = k$ of the seven English semantic networks. (a) Network *'Has-A'*, (b) Network *'Is-A'*, (c) Network *'Part-Of'*, (d) Network *'Related-To'*, (e) Network *'Union'*, (f) Network *'Antonym'*, (g) Network *'Synonym'*. The circle data points are the original average local clustering coefficients of nodes with degree $d_i = k$, triangles represent data after logarithmic binning, and squares show the average clustering coefficient of nodes with degree $d_i = k$ (logarithmically binned) in the randomized networks.

Descriptive statistics of the semantic networks from different languages

This section shows the descriptive statistics of semantic networks from the eleven languages. Each property is compared among the seven networks for the eleven languages.

Language classifications

Table S5 shows the typological and genetic classifications of the eleven considered languages.

Genetic \ Typological	***Inflecting***	***Isolating***	***Agglutinating***
Italic	Spanish, French, Italian, Portuguese		
Germanic	English, Dutch, German		
Balto-Slavic	Russian		
Transeurasian			Japanese
Sino-Tibetan		Chinese	
Uralic			Finnish

Table S5. Genetic and typological language classifications of the eleven languages.

Overview statistics of semantic networks from different languages

Table S6 shows the number of nodes of each semantic network in the eleven different languages. A blank element in the table indicates that the network does not exist, *i.e.*, a relation is not available in that language.

Network	***Has-A***	***Is-A***	***Part-Of***	***Related-To***	***Union***	***Antonym***	***Synonym***
English	1,664	140,024	7,562	571,079	650,079	5,912	53,279
French		17,519	2,832	1,289,083	1,296,622	1,361	20,144
Italian		2,663	9	36,295	46,468	13	1,580
German		113,301	5	100,737	172,147	187	43,072
Spanish		255	11	12,094	22,861	15	3,491
Russian		557	3	20,268	25,887	12	1,148
Portuguese		3,341	15	5,929	11,426	17	6,421
Dutch		191	53	303	1,418	111	11,964
Japanese	38	40,256	7,230	7,200	43,286	20	230
Finnish		76	12	4,483	6,958	24	1,569
Chinese	6,355	10,073	3,417	3,163	17,128	4	17

Table S6. Number of nodes N in the LCCs of the semantic networks from the eleven different languages extracted from ConceptNet. A blank element indicates the corresponding network is not available. The ‘Union’ network is the union of four networks (‘Has-A’, ‘Is-A’, ‘Part-Of’ and ‘Related-To’). Because we display the LCC sizes, for some ‘Union’ networks, the number of nodes exceeds the sum of the sizes of its four constituent networks.

Average degree in the LCCs of semantic networks from different languages

Network	***Has-A***	***Is-A***	***Part-Of***	***Related-To***	***Union***	***Antonym***	***Synonym***
English	2.21	3.05	2.44	5.60	5.55	2.70	3.03
French		2.64	2.51	3.44	3.46	2.45	2.81
Italian		2.86	2.89	2.20	2.27	1.85	2.54
German		2.75	1.60	4.77	4.53	2.16	3.57
Spanish		2.45	2.73	2.13	2.13	1.87	2.57
Russian		2.23	1.33	4.14	3.88	1.83	2.26
Portuguese		2.24	2.67	2.49	2.65	2.00	2.84
Dutch		4.68	4.98	2.30	2.69	2.11	3.53
Japanese	2.89	4.42	4.11	4.34	4.79	2.00	2.73
Finnish		1.97	1.83	2.30	2.26	1.92	2.24
Chinese	3.58	3.02	3.36	4.06	3.78	1.50	2.24

Table S7. Average degree $E[D]$ in the LCCs of the semantic networks from the eleven different languages extracted from ConceptNet. A blank element indicates the corresponding network is unavailable.

Estimated power-law exponents for semantic networks from different languages

Table S8 lists the estimated power-law exponents $\hat{\gamma}$ for each semantic network in the eleven languages. We consider a network to not have a power-law degree distribution if it is not or hardly power-law according to the method of Voitalov *et al.*[4].

Network	γ	***Has-A***	***Is-A***	***Part-Of***	***Related-To***	***Union***	***Antonym***	***Synonym***
English	$\hat{\gamma}^{Slope}$	2.3	2.3	2.4	2.4	2.4	×	×
	$\hat{\gamma}^{Hill}$	2.3	2.3	2.5	2.3	2.3	×	×
	$\hat{\gamma}^{Mom}$	2.5	2.3	2.6	2.2	2.2	×	×
	$\hat{\gamma}^{Kern}$	2.6	2.3	2.7	2.1	2.1	×	×
French	$\hat{\gamma}^{Slope}$		2.4	2.3	×	×	2.7	3.1
	$\hat{\gamma}^{Hill}$		2.5	2.5	×	×	3.3	3.6
	$\hat{\gamma}^{Mom}$		2.5	2.6	×	×	4.5	3.9
	$\hat{\gamma}^{Kern}$		2.6	2.6	×	×	4.2	4.8
Italian	$\hat{\gamma}^{Slope}$		2.3		2.6	2.6		×
	$\hat{\gamma}^{Hill}$		2.8		2.4	2.4		×
	$\hat{\gamma}^{Mom}$		2.2		2.4	2.5		×
	$\hat{\gamma}^{Kern}$		2.3		2.5	2.6		×
German	$\hat{\gamma}^{Slope}$		2.5		2.6	2.5		3.1
	$\hat{\gamma}^{Hill}$		2.2		2.7	2.9		3.6
	$\hat{\gamma}^{Mom}$		3.3		2.7	2.9		3.7
	$\hat{\gamma}^{Kern}$		2.3		2.9	2.6		3.9
Spanish	$\hat{\gamma}^{Slope}$				×	×		×
	$\hat{\gamma}^{Hill}$				×	×		×
	$\hat{\gamma}^{Mom}$				×	×		×
	$\hat{\gamma}^{Kern}$				×	×		×
Russian	$\hat{\gamma}^{Slope}$				×	×		×
	$\hat{\gamma}^{Hill}$				×	×		×
	$\hat{\gamma}^{Mom}$				×	×		×
	$\hat{\gamma}^{Kern}$				×	×		×
Portuguese	$\hat{\gamma}^{Slope}$		2.6		2.4	2.5		×
	$\hat{\gamma}^{Hill}$		2.8		2.6	2.8		×
	$\hat{\gamma}^{Mom}$		2.6		2.1	2.4		×
	$\hat{\gamma}^{Kern}$		2.6		2.9	2.7		×
Dutch	$\hat{\gamma}^{Slope}$					2.2		×
	$\hat{\gamma}^{Hill}$					2.8		×
	$\hat{\gamma}^{Mom}$					3.1		×
	$\hat{\gamma}^{Kern}$					3.5		×
Japanese	$\hat{\gamma}^{Slope}$		2.4	2.3	2.2	2.3		
	$\hat{\gamma}^{Hill}$		2.6	2.9	4.9	2.6		
	$\hat{\gamma}^{Mom}$		2.6	2.9	2.4	2.7		
	$\hat{\gamma}^{Kern}$		2.6	2.6	2.6	2.6		
Finnish	$\hat{\gamma}^{Slope}$				×	×		×
	$\hat{\gamma}^{Hill}$				×	×		×
	$\hat{\gamma}^{Mom}$				×	×		×
	$\hat{\gamma}^{Kern}$				×	×		×
Chinese	$\hat{\gamma}^{Slope}$	2.5	2.3	2.7	1.9	2.3		
	$\hat{\gamma}^{Hill}$	3.4	2.4	2.3	2.7	4.3		
	$\hat{\gamma}^{Mom}$	3.8	2.4	2.4	1.9	2.4		
	$\hat{\gamma}^{Kern}$	2.7	2.3	2.3	2.3	2.5		

Table S8. Estimated power-law exponents $\hat{\gamma}$ for the LCCs of the semantic networks in different languages. A blank element indicates the corresponding network is either unavailable or the number of nodes $N < 1000$. A cross (×) indicates that the degree sequence of that network is not or hardly power-law.

Examples of words in the peak and their neighoring words in the Spanish 'Related-To' network

Peak word	***Translation***	***Neighbors***
cenar	to dine	cená, cenábamos, cenáculo, cenáis, cenáramos, cenáremos, ...
viajar	to travel	viaja, viajaba, viajabais, viajaban, viajabas, viajad, viajado, ...
pasear	to walk	pasea, paseaba, paseabais, paseaban, paseabas, pasead, ...
reparar	to repair	repararais, repararan, repararas, repararéis, repararemos, ...
comparar	to compare	comprar, comparaba, comparabais, comparaban, comparabas, ...

Table S9. Examples of words in the peak and their neighoring words in the Spanish 'Related-To' network.

Percentages of POS tags among peak words and in the LCCs of 'Related-To' networks in four inflecting languages

Percentage (%)	**French**		**Spanish**		**Portuguese**		**Finnish**	
	LCC	Peak	LCC	Peak	LCC	Peak	LCC	Peak
POS tagged	98.71	98.66	92.72	77.84	67.60	60.00	81.37	64.13
Verb	68.90	89.97	87.62	98.44	32.56	100.00	11.40	11.36
Noun	19.21	7.14	9.20	1.56	51.96	0	77.96	84.09
Adjective	11.53	2.75	2.89	0	14.60	0	7.17	4.55
Adverb	0.36	0.15	0.29	0	0.88	0	3.47	0

Table S10. Percentages of POS tags among peak words and in the LCCs of the 'Related-To' networks of four inflecting languages.

Percentage of verbs and nouns among the neighbors of peak words of the LCC of 'Related-To' networks of four inflecting languages

Percentage (%)	**French**		**Spanish**		**Portuguese**		**Finnish**	
	Mean	SD	Mean	SD	Mean	SD	Mean	SD
POS tagged	97.39	0.88	96.96	1.73	97.74	0.75	93.72	4.45
Verb	87.26	25.85	97.24	2.59	99.23	0.94	3.86	14.64
Noun	9.34	20.08	2.07	2.15	0.77	0.94	89.67	26.50

Table S11. The mean and Standard Deviation (SD) percentage of verbs and nouns in the neighbors of peak words of the LCC of network 'Related-To' in four inflecting languages.

Node merging procedure

First, we extract the network 'Form-Of' in the same way as for all other networks. Then we treat the merged group of words as a single word in the 'Related-To' network in the same language. Next, we calculate the number of nodes with degree k in the new 'Related-To' network. Finally, we plot the densities of the degree distributions of French, Spanish, Portuguese and Finnish networks.

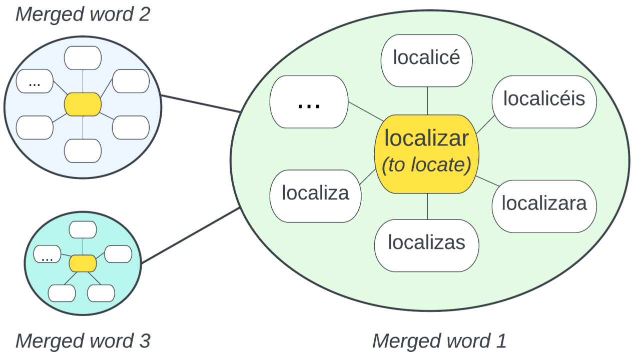

Figure S5. Illustration of the merging of words in the 'Related-To' network. After merging a root word and its neighbors, all words in a circle are seen as a single word.

The percentages of matched words among the peak words for the LCCs of the 'Related-To' networks in four languages

Percentage	French	Spanish	Portuguese	Finnish
Percentage of peak words covered by 'Form-Of'	33.72%	100%	60.00%	91.30%
Percentage of neighbors of peak words covered by 'Form-Of'	17.38%	97.76%	55.47%	45.08%

Table S12. The percentages of matched words among the peak words of the LCCs of the 'Related-To' networks in four languages.

The number of grammatical variations in French, Spanish, Portuguese and Finnish

Language	*Grammatical variations m*	k_{min}	k_{max}
French	42	36	51
Spanish	54	45	61
Portuguese	54	53	53
Finnish	30	25	35

Table S13. The maximum number of grammatical variations m for the grammatical rule of interest in French, Spanish, Portuguese and Finnish. The minimum and maximum degree k_{min} and k_{max} where the peak starts and ends in the densities of the degree distributions of the 'Related-To' networks are included for comparison.

Structural similarity and complementarity coefficients

For the convenience of the reader, here we summarize the main components of the framework for computing structural similarity and complementarity coefficients by Talaga and Nowak[6].

Structural coefficients

Similarity-based networks are rich in triangles because of the triangle closure principle. The clustering coefficient is a classic measure of the density of triangles in a network. However, we cannot simply compare the number of triangles and quadrangles between two networks, because these networks have different sizes and degree distributions. We need to reliably calculate the statistics of triangles and quadrangles of a network to quantify similarity and complementarity. To this end, we rely on a recent work on complementarity[6]. The structural similarity coefficient is a weighted average of two clustering coefficients based on head and wedge triples (Figs. S6c and S6b). Analogous to the clustering coefficient, we can use structural complementarity measures based on quadrangle closure rules (Fig. S6d). Similarly, the structural complementarity coefficient is a weighted average of two coefficients based on head and wedge quadrangles (Figs. S6f and S6e). Here, we summarize the

main components of procedures of calculating the structural similarity coefficient and complementarity coefficient of a network G.

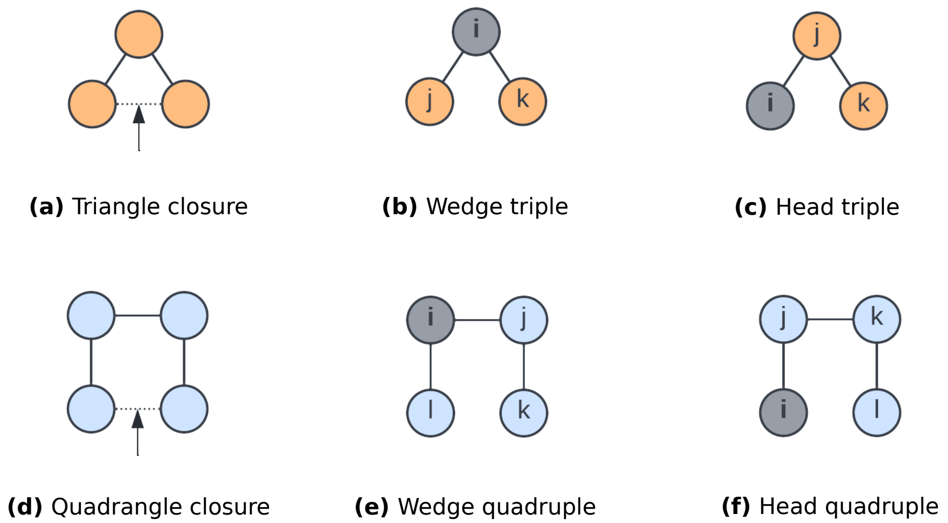

Figure S6. Quadrangle and quadruples in comparison with triangle and triples. Wedge and head triples (or quadruples) are different at where node i is centered. Node i in a wedge triple (b) is centered in the middle, while i in a head triple (c) is centered at the beginning. Similarly, node i in a wedge quadruple (e) is centered at the second location, while i is at the beginning of a head quadruple (f).

Structural similarity coefficient

The structural similarity coefficient s_i generalizes the local clustering and closure coefficients. The local clustering coefficient s_i^W of a node i is the classic clustering coefficient. It is defined as the fraction of triples centered at i which can be closed to form a triangle,

$$s_i^W = \frac{2T_i}{t_i^W} = \frac{\sum_{j,k} a_{ij} a_{ik} a_{jk}}{d_i (d_i - 1)}, \tag{3}$$

where T_i is the number of triangles including i and t_i^W is the number of wedge triples (Fig. S6b), or 2-paths with node i in the middle, *e.g.*, (j, i, k). The definition of the local closure coefficient[7] is given as follows

$$s_i^H = \frac{2T_i}{t_i^H} = \frac{\sum_{j,k} a_{ij} a_{ik} a_{jk}}{\sum_j a_{ij} (d_j - 1)}, \tag{4}$$

where t_i^H is the number of head triples (Fig. S6c), *i.e.*, 2-paths starting from node i, such as (i, j, k). Both s_i^W and s_i^H are bounded in the range $[0, 1]$, but they capture different parts of the spectrum of similarity-driven structures[6].

Combining the weighted average of these two coefficients results in a more comprehensive measure of local structure, the *structural similarity coefficient*[6], which captures the full spectrum of structural similarity. It is defined as

$$s_i = \frac{4T_i}{t_i^W + t_i^H} = \frac{t_i^W s_i^W + t_i^H s_i^H}{t_i^W + t_i^H}. \tag{5}$$

The coefficient $s_i = 1$ only if node i is in a fully connected network.

The structural similarity coefficient of a whole network G is then the average over all nodes

$$s(G) = \frac{1}{N} \sum_{i=1}^{N} s_i. \tag{6}$$

Structural complementarity coefficient

Analogously, the local quadruples clustering coefficient at node i is defined as the fraction of closed quadruples with i at the second position[6]

$$c_i^W = \frac{2Q_i}{q_i^W} = \frac{\sum_{j\neq i} a_{ij} \sum_{k\neq i,j} a_{ik} \left(1-a_{jk}\right) \sum_{l\neq i,j,k} a_{kl} a_{jl} \left(1-a_{il}\right)}{\sum_j a_{ij} \left[\left(d_i-1\right)\left(d_j-1\right)-n_{ij}\right]}, \tag{7}$$

where Q_i represents the number of quadrangles contain that node i and q_i^W is the number of wedge quadruples (Fig. S6e), or 3-paths with i at the second node, *e.g.*, (l,i,j,k). Similarly, the local quadruples closure coefficient of a node i calculates the percentage of closed quadruples beginning at i

$$c_i^H = \frac{2Q_i}{q_i^H} = \frac{\sum_{j\neq i} a_{ij} \sum_{k\neq i,j} a_{ik} \left(1-a_{jk}\right) \sum_{l\neq i,j,k} a_{kl} a_{jl} \left(1-a_{il}\right)}{\sum_{j\neq i} a_{ij} \sum_{k\neq i,j} a_{jk} \left(d_k-1-a_{ik}\right)}, \tag{8}$$

where q_i^H is the number of head quadruples originating from node i (Fig. S6f).

Finally, the *structural complementarity coefficient* is constructed as the weighted average of the local quadruples clustering and closure coefficients[6]

$$c_i = \frac{4Q_i}{q_i^W + q_i^H} = \frac{q_i^W c_i^W + q_i^H c_i^H}{q_i^W + q_i^H}. \tag{9}$$

The structural complementarity coefficient $c_i \in [0,1]$, which is proven to be a more general measure than using only c_i^W or c_i^H[6]. The maximum $c_i = 1$ happens only if node i belongs to a fully connected bipartite graph. In a bipartite graph, nodes are divided into two groups, and connections are only formed between groups but not within the same group.

The structural complementarity coefficient of a whole network G is then the average of all nodes:

$$c(G) = \frac{1}{N}\sum_{i=1}^{N} c_i. \tag{10}$$

Table S14 lists the procedures of how we compute the structural similarity and complementarity coefficients to quantify the density of triangles and quadrangles in a network G, respectively.

Procedure	Structural coefficients	Network G	
		**Similarity** (△)	**Complementarity** (□)
Step 1	Wedge triple/quadruple	s_i^W, Eq. 3	c_i^W, Eq. 7
	Head triple/quadruple	s_i^H, Eq. 4	c_i^H, Eq. 8
Step 2	Node-wise	s_i, Eq. 5	c_i, Eq. 9
Step 3	Network-wise	$s(G) = \frac{1}{N}\sum_{i=1}^{N} s_i$, Eq. 6	$c(G) = \frac{1}{N}\sum_{i=1}^{N} c_i$, Eq. 10
Step 4	Calibrated Network-wise	$\mathscr{C}(s)_G = \frac{1}{R}\sum_{i=1}^{R} \log\frac{s(G)}{s(G_i)}$	$\mathscr{C}(c)_G = \frac{1}{R}\sum_{i=1}^{R} \log\frac{c(G)}{c(G_i)}$

Table S14. The procedure of calculating the structural similarity coefficient and complementarity coefficient of a network G. The calibrated structural coefficients in step 4 are obtained by taking the average log ratio of a network-wise coefficient over the coefficients of sampled networks G_i, see Eq. 11.

Calibration

This section presents the configuration model used to calibrate structural coefficients of semantic networks. The details of the calibration process are provided as well.

Undirected Binary Configuration Model

In this paper, we utilize the Undirected Binary Configuration Model (UBCM)[8] to calibrate the structural coefficients. The UBCM generates a maximum entropy probability distribution over a network with the constraints of an expected degree sequence. It is suitable for undirected and unweighted networks. The resulting maximum entropy distributions are maximally unbiased with respect to any other property[9].

Calibration of structural coefficients

First of all, we calculate one structural coefficient (similarity or complementarity) of a given network G. We denote this coefficient as $x(G)$. Second, we sample R randomized copies G_i's of the given network from the configuration model. Then, we calculate the structural coefficient $x(G_i)$ for each sampled network. At last, we take the average log ratio of $x(G)$ and $x(G_i)$'s. As a result, the calibrated coefficient $\mathscr{C}_G(x)$ based on R samples from the configuration model is obtained as follows[6]

$$\mathscr{C}(x)_G = \frac{1}{R}\sum_{i=1}^{R} \log \frac{x(G)}{x(G_i)}. \tag{11}$$

The calibrated structural coefficient can be less than, equal to or larger than zero. Consider the calibrated structural similarity coefficient $\mathscr{C}_G(s)$ for example:

- $\mathscr{C}_G(s) < 0$, the structural similarity coefficient $s(G)$ is smaller than $s(G_i)$ of random networks.
- $\mathscr{C}_G(s) = 0$, the structural similarity coefficient is comparable to the ones in random networks.
- $\mathscr{C}_G(s) > 0$, the structural similarity coefficient is larger than in random networks.

We do not compute the structural coefficients for networks that have less than 100 nodes, because there is a high chance that there exist no triangles or quadrangles in the sampled networks and the structural coefficient $x(G_i) = 0$, in that case. When $x(G_i) = 0$, Eq. 11 is undefined.

Since the runtime of the algorithm depends on the size of a network and the choice of the number of randomized networks R, we do not compute the structural coefficients for the two largest networks, the French 'Related-To' and 'Union' networks with $N > 1,200,000$ each, as the computation time would be infeasible. We use $R = 500$ for most networks and for the remaining two largest networks, English 'Related-To' and 'Union', we set $R = 100$ to avoid long computation time.

References

1. Speer, R., Chin, J. & Havasi, C. Conceptnet 5.5: An open multilingual graph of general knowledge. In *Proceedings of the Thirty-First AAAI Conference on Artificial Intelligence*, AAAI'17, 4444–4451 (AAAI Press, 2017).
2. Speer, R. Relations in ConceptNet5. *ConceptNet 5 Wiki* https://github.com/commonsense/conceptnet5/wiki/Relations (2019).
3. White, E., Enquist, B. & Green, J. On estimating the exponent of power-law frequency distributions. *Ecology* **89**, 905–12, DOI: 10.1890/07-1288.1 (2008).
4. Voitalov, I., van der Hoorn, P., van der Hofstad, R. & Krioukov, D. Scale-free networks well done. *Phys. Rev. Res.* **1**, 033034 (2019).
5. Voitalov, I. Tail index estimation for degree sequences of complex networks. https://github.com/ivanvoitalov/tailestimation (2019).
6. Talaga, S. & Nowak, A. Structural measures of similarity and complementarity in complex networks. *Sci. Rep.* **12**, 16580 (2022).
7. Yin, H., Benson, A. R. & Leskovec, J. The local closure coefficient: A new perspective on network clustering. In *Proceedings of the Twelfth ACM International Conference on Web Search and Data Mining*, 303–311 (2019).
8. Vallarano, N. *et al.* Fast and scalable likelihood maximization for exponential random graph models with local constraints. *Sci. Rep.* **11**, 1–33 (2021).
9. Squartini, T., Mastrandrea, R. & Garlaschelli, D. Unbiased sampling of network ensembles. *New J. Phys.* **17**, 023052 (2015).
